# 

**Published:** 1820-09

**Authors:** 


### AUTUMNAL LECTURES.*

### *Anatomy and Physiology.*

At the School of Great Windmill-street; by Mr. Wilson and Mr. Charles Bell. The Demonstrations by Mr. Shaw. To commence on the 2d of October.

Mr. Carpue will commence his Lectures on the 2d of October, at his Theatre, 50, Dean-street, Soho.

Mr. Edward Grainger, on Tuesday the 3d of October, at his Rooms, 13, St. Saviour's Church-yard, Southwark.

Mr. Taunton will begin his Lectures on the 7th of October, at eight o'clock in the evening, at his Theatre in Hatton Garden.

Mr. Guthrie on Diseases of the Eye.

### *On Medicine.*

At the Medical Theatre, No. 42, Great Windmill-street: On Physiology and the Practice of Physic, by Richard Harrison, M.D. F.L.S. Fellow of the Royal College of Physicians, &c. will commence at nine o'clock on Monday morning, the 2d of October. On the Materia Medica, including the doctrines of Pharmacy and Therapeutics, by John Ayrton Paris, M.D. F.L.S. Professor of the Materia Medica to the College of Physicians, will commence at ten o'clock on Monday morning, the 2d of October. The Lectures will be continued every Monday, Wednesday, and Friday, at the above hours.

Dr. Cloves and Dr. Gregory will begin their winter course of Lectures on the Theory and Practice of Physic, and on the Materia Medica, on Monday, October 9th, at nine o'clock in the morning, at No. 14, Old Burlington-street.

* We insert this List of Lectures as a matter of intelligence, conformable with the plan of our Journal; and we shall insert, annually, at the commencement of the autumnal season, any information of this kind that may be communicated to us; but it must be conveyed in general terms, as above stated. Those of our readers who desire that more particular notices of their Lectures should appear with this Journal, must send them to the Publisher, not the Editor. Such notices are considered by the tax-officers as *advertisements;* they must, therefore, be placed on the Wrapper, and the duty on them paid to the Publisher when they are transmitted to him.

Dr. Clutterbuck on the Theory and Practice of Physic, Materia Medica, and Chemistry.

*Midwifery.*

Mr. Clarke and Mr. Blagden will begin their Lectures on Midwifery and the Diseases of Women and Children, on Monday, October 9th, at No. 10, Saville-row.

Mr. Davies on the Theory and Practice of Midwifery, and on the Diseases of Women and Children.

*Medical School, St. Bartholomew's Hospital.*—The following courses of Lectures will be commenced on Monday, October 2d. On the Theory and Practice of Medicine, and on Chemistry and Materia Medica, by Dr. Hue; on Anatomy and Physiology, and on the Theory and Practice of Surgery, by Mr. Abernethy; on Midwifery, by Dr. Gooch; Practical Anatomy, with Demonstrations, by Mr. Stanley.

*Middlesex Hospital.*—The Medical Lectures will commence on Monday, October the 9th. Theory and Practice of Physic, by Dr. Southey; Chemistry, by Dr. Ashburner; Materia Medica, by Dr. Southey and Dr. Ashburner; Midwifery, and the Diseases of Women and Children, by Dr. Merriman and Dr. Ley.

*St. George's Medical and Chirurgical School.*—Dr. Pearson on the Practice of Physic, and on Therapeutics and Materia Medica; Professor Brande on Chemistry; B. C. Brodie, F.R.S. on Surgery; Sir Everard Home, Bart. on Surgery.

---

### REPORT OF DISEASES.

### MONTHLY CATALOGUE OF MEDICAL BOOKS.

Elements of the Theory and Practice of Physic, designed for the Use of Students. Part I. including the Symptoms, Pathology, and Treatment, of Acute Diseases. By George Gregory, M.D. 1 vol. 8vo.

A Synopsis of the various kinds of difficult Parturition, with Practical Remarks on the Management of Labours. Third Edition, with considerable Additions, and an Appendix of illustrative Cases and Tables. By Samuel Merriman, M.D. 8vo. 12s.

An Address to Dropsical Patients, including such Diseases, and proposing a method of Treatment upon new and successful Principles, confirmed by some most astonishing Cures in several Cases which were complicated with Diseased Liver, Jaundice, and other alarming Symptoms. By D. Coley, Member of the Royal College of Surgeons. 1s.

Numerous Cases illustrative of the Efficacy of the Hydrocyanic or Prussic Acid in Affection of the Stomach; with a Report upon its Powers in Pectoral and other Diseases in which it has been already recommended. By John Elliotson, M.D. &c. 8vo. 5s. 6d.

Dentiste de la Jeunesse; or, the Way to have Sound and Beautiful Teeth. Translated from the French of Duval, by J. Atkinson, Surgeon-Dentist. 8vo. 7s.

Atkinson's Compendium of Ornithology. 8vo. 8s.

[MESSRS. HIGHLEY AND SON.]

